# Treatment of intestinal schistosomiasis in Ugandan preschool children: best diagnosis, treatment efficacy and side-effects, and an extended praziquantel dosing pole

**DOI:** 10.1016/j.inhe.2010.02.003

**Published:** 2010-06

**Authors:** José Carlos Sousa-Figueiredo, Joyce Pleasant, Matthew Day, Martha Betson, David Rollinson, Antonio Montresor, Francis Kazibwe, Narcis B. Kabatereine, J. Russell Stothard

**Affiliations:** a WHO Collaborating Centre Schistosomiasis, Wolfson Wellcome Biomedical Laboratories, Department of Zoology, Natural History Museum, London, SW7 5BD, United Kingdom; b Department of Infectious and Tropical Diseases, London School of Hygiene & Tropical Medicine, London, WC1E 7HT, United Kingdom; c Faculty of Medicine, University of Dundee, Nethergate, Dundee, DD1 4HH, United Kingdom; d Department of Control of Neglected Tropical Diseases, World Health Organization, 20 Avenue Appia, 1211 Geneva 27, Switzerland; e Vector Control Division, Ministry of Health, P.O. Box 1661, Kampala, Uganda

**Keywords:** Schistosomiasis, national control programme, preschoolaged children (PSAC), deworming, guidelines

## Abstract

The Ugandan national control programme for schistosomiasis has no clear policy for inclusion of preschool-children (≤5 years old) children. To re-balance this health inequality, we sought to identify best diagnosis of intestinal schistosomiasis, observe treatment safety and efficacy of praziquantel (PZQ), and extend the current WHO dose pole for chemotherapy. We examined and treated 363 preschool children from shoreline villages of Lakes Albert and Victoria, and found that 62·3% (CI_95_ 57·1–67·3) of the children were confirmed to have intestinal schistosomiasis. One day after treatment, children were reported as having headaches (3·6%), vomiting (9·4%), diarrhoea (10·9%) and urticaria/rash (8·9%) with amelioration at 21-day follow-up, where the parasitological cure rate was found to be 100·0%. Height and weight data were collected from a further 3303 preschool children to establish and validate an extended PZQ dose pole that now includes two new height-intervals: 60–84 cm for one-half tablet and 84–99 cm for three-quarter tablet divisions; which would result in 97·6% of children receiving an acceptable dose (30–60 mg/kg). To conclude, preschool children in lakeshore communities of Uganda are at significant risk of intestinal schistosomiasis; we now strongly advocate for their immediate inclusion within the national control programme to eliminate this health inequity.

## 1 Introduction

Schistosomiasis is considered a neglected tropical disease (NTD) and affects around 200 million people worldwide, many of whom develop severe and permanent disabilities.^1^, ^2^ Control of the infection has gained much international interest and political commitment since 2000, when the United Nations member states and 23 international organizations agreed on the eight Millennium Development Goals (MDGs). The World Health Organization (WHO) subsequently advocated that control of schistosomiasis contributes to the achievement of the MDGs, and in 2001 the World Health Assembly endorsed resolution 54·19 which recommends regular de-worming of school-aged children at risk of infection (http://www.who.int/inf-pr-2001/en/pr2001WHA-6.html).^3^, ^4^, ^5^In the fight against these diseases of poverty, preventive chemotherapy campaigns are now the front-line intervention, administering safe, efficacious and low-cost anthelminthics, i.e. praziquantel (PZQ) for schistosomiasis and albendazole (ALB) for soil-transmitted helminthiasis (STHs).^6^ In the past decade, several campaigns have been implemented throughout sub-Saharan Africa targeting school children (six to 15 years old) and/or adults (over 15 years old) in high-risk occupational groups (e.g. fishermen).^7^, ^8^ Since 2003, more than nine million school-aged children have been treated for schistosomiasis in Uganda, and yet, as an unforeseen consequence of current guidelines, young children (≤ six years old) have been consistently overlooked.^8^, ^9^ For example, only recently has WHO policy categorised PZQ as a safe drug for children as young as four years of age, an age-limit not yet reflected in the drug package insert of most drug producers, even though historical and recent evidence suggests that risk of infection starts even earlier.^10^, ^11^, ^12^, ^13^, ^14^, ^15^, ^16^, ^17^, ^18^ Moreover, the current WHO dose pole for PZQ administration is only applicable to children taller than 94 cm, typically aged between four and five years, and the risk of choking due to the size, shape and palatability of PZQ tablets has dissuaded mass drug administration initiatives to include smaller children in their targeted populations.^19^ Nevertheless, given the young child's disease and enhanced risk for developing morbidity in early childhood, this current health inequity should not persist.^14^, ^20^, ^21^, ^22^

Thus to develop better national policies within Uganda, and with a view to refining international guidelines, we aimed to provide detailed evidence on occurrence of intestinal schistosomiasis in very young children (≤ six years of age), to observe the efficacy and safety of PZQ in this age-class and extend the current dose pole to facilitate the allocation of treatments within mass drug administration initiatives.

## 2 Methods

### 2.1 Study areas and populations

**Figure 1:**
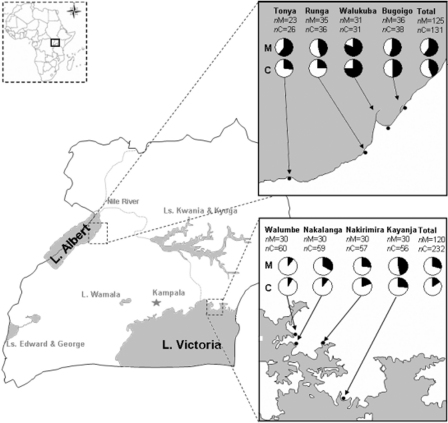
Schematic map of Uganda, with its major water bodies (in grey). Inset are a map of Africa (top left: Uganda is highlighted) as well as detailed maps of the study areas in Lake Albert (top right) and Victoria (bottom right), depicting the prevalence of intestinal schistosomiasis in both mothers (*n*M) and children (*n*C) from different villages. The area of the dark sector in the pie chart is proportional to the prevalence of positives for egg-patent infection. Refer to Table 1 for prevalence values and CI_95_.

Two targeted epidemiological surveys aimed at detecting intestinal schistosomiasis in preschool children were undertaken on separate occasions in areas highly endemic for intestinal schistosomiasis.^23^ The first survey took place in July 2007 in the Lake Albert region (Buliisa District) and examined 125 mothers and their 131 preschool children (≤six years of age). The second survey was conducted in January/February 2009 in the Lake Victoria region (Mayuge District) where 120 mothers and their 232 preschool children were examined. Prior to enrolment, village-wide community sensitisation to the study's objectives was conducted by liaison with local health workers. Families were then recruited foremost upon preliminary informed assent and availability to attend the two-day walk-in survey clinic to maximise provision of duplicate faecal samples, enhancing the opportunity for more precise detection of infections. The lake systems and eight study villages of Tonya, Runga, Walukuba and Bugoigo (along Lake Albert shoreline) and Walumbe, Nakalanga, Nakirimira and Kayanja (along Lake Victoria shoreline) are shown in Figure 1.

### 2.2 Case-history questionnaires

As young children were to be examined it was essential to include each child's mother for assistance in provision of stool and urine samples from her child. Each mother was also asked directly, or on behalf of her child, 27 questions (in Lake Albert) or 24 (in Lake Victoria) recording: demographic information, general socio-economic indicators, access to health care, general self-reported health conditions and other variables (e.g. water contact behaviours and access to medications). Upon the basis of preliminary analysis, three questions used in Lake Albert were subsequently deemed superfluous and therefore omitted from the Lake Victoria survey. Copies of the case-history questionnaires are available upon request from the corresponding author.

### 2.3 Detection of schistosomiasis

Parasitological diagnosis of *Schistosoma mansoni* was performed using two consecutive day stool samples and double Kato-Katz (KK) smears prepared for each child and mother.^24^ Results were expressed as eggs per gram of faeces (epg) and infection intensities of *S. mansoni* were categorised as follows: 1–100 epg as light, 101–400 epg as medium and >400 epg as heavy infections. Finger-prick blood (∼50 μl) was taken from mother and child to obtain serum to ascertain schistosomiasis status using a commercially available soluble egg antigen enzyme-linked immunosorbent assay (SEA-ELISA; IVD Research Inc., Calsbad, USA).^25^ A single urine sample, taken from each mother and child, provided a 50 μl aliquot for testing of the presence of schistosome circulating cathodic antigen (CCA) with a commercially available immuno-chromatographic dipstick (Rapid Medical Diagnostics, Pretoria, RSA), a rapid diagnostic test for intestinal schistosomiasis.^26^

### 2.4 Efficacy of treatment with PZQ and side-effects

Irrespective of infection status and to mimic mass drug administration conditions during the investigative epidemiological surveys, all mothers and children received standard doses of PZQ (CIPLA, Mumbai, India) calculated by weight. In addition ALB (GSK, Uxbridge, UK) was provided.^18^ For younger/smaller children (<2 year olds), the PZQ tablets were crushed and mixed with orange juice and sugar before administration (spoon-feeding), with the help of the mother, along with a chewable orange flavoured half-tablet of ALB.

Efficacy of PZQ upon intestinal schistosomiasis was assessed 21 days after treatment with a follow-up visit using parasite detection methods as described above. For logistical limitations, this section of the survey was only conducted in the four villages of Lake Victoria. Two different cure rates (CR: the percentage of the infected population negative for infection after drug treatment) were calculated, the first using data from microscopy visualising egg-patent infections and second using urine CCA-dipsticks. Egg reduction rate (ERR: the percentage reduction in egg intensity as measured by faecal epg after drug treatment) was calculated.^27^

As part of a large-scale de-worming exercise in Bugoigo and Walukuba villages (Lake Albert), a follow-up of 1122 preschool children was performed 24 h after treatment, where mothers were asked to report whether their children needed any medical assistance or had experienced any of the following symptoms since treatment: dizziness, headache, sleepiness, fatigue, vertigo, abdominal pain, cramps, nausea, vomiting, diarrhoea, bloody stools, lower back pain and urticaria/rash. Due to this exercise, a more formalised approach was taken in Lake Victoria (193 children) to assess putative side-effects to PZQ. Once again, mothers responded on behalf of their children as to whether their child experienced any of the stated symptoms prior to treatment, at 24 hours and 21 days after-treatment. Any symptom putatively associated with PZQ treatment was assessed by adjusting follow-up levels to pre-treatment levels.

### 2.5 PZQ dose pole

During the two mass treatment exercises undertaken in conjunction with the epidemiological surveys, height and weight measurements were collected from 2093 preschool children (1144 children from Lake Albert and 949 children from Lake Victoria). Randomly selected data points from Lake Albert (*n* = 572) and Lake Victoria (*n* = 474) were compiled and used to establish a correlation between height and weight of preschool children.^28^, ^29^ From this correlation, two new height thresholds were established, corresponding to one-half and three-quarters of one 600 mg tablet of PZQ, and the current threshold for one tablet of PZQ on the WHO dose pole was amended.^19^ The extended dose pole was then theoretically tested in three phases: firstly, on the remaining data from the same cross-sectional survey (572 children from Lake Albert and 475 children from Lake Victoria) to assess its applicability on an equivalent population; secondly, on data from a different Ugandan population surveyed between April and July 2009 in the same districts but from different villages (573 children from Lake Albert and 637 children from Lake Victoria); and thirdly, on data from 470 Zanzibari preschool children to assess the new dose pole's applicability in another schistosomiasis*-*endemic country.^13^ On all occasions, height and weight measurements were performed to precisions of 0·1 cm and 0·1 kg, respectively. A safe and efficacious (i.e. acceptable) dose of PZQ was defined as being between 30–60 mg/kg, and an optimal dose as being between 40–60 mg/kg.^19^

### 2.6 Statistical analysis

Data were collected from each individual using pro-forma data sheets, which were then entered using EpiData™ (The EpiData Association, Odense, Denmark) and Microsoft Excel^®^ spreadsheet software. The data thus collated were analysed using the R statistical package^®^ v 2·8·1 (The R Foundation for Statistical Computing, Vienna, Austria).^30^ For prevalence values, 95% confidence intervals (CI_95_) were estimated using the exact method.^31^ Prevalence comparisons were performed using (one-tailed) Fisher's exact modification of the 2 × 2 chi-squared test.^32^ For infection intensity values, the geometric mean of Williams, GM_W_, was chosen as the measure of central tendency due to the typical over-dispersion present in this type of data, and CI_95_ values for GM_W_ were estimated according to Kirkwood and Stern.^33^, ^34^

Univariate logistic regression was carried out to ascertain risk factors associated with intestinal schistosomiasis in children and their mothers. Within village intra-correlation in the data was accounted for using a generalized linear mixed model with multivariate normal random effects (the random-effects of village in our case), with penalized quasi-likelihood (function glmmPQL in R).^35^ For each variable, odds ratio (OR) and *P*-values were calculated, and a *P*-value < 0·05 was considered indicative of statistical significance.

### 2.7 Ethical approval, informed consents

The Ugandan National Council of Science and Technology and the London School of Hygiene and Tropical Medicine, UK, granted ethical approvals for these studies (application nos. LSHTM 06·45 and LSHTM 5538·09). All mothers were sensitized by community leaders (Local Chairman, level 1), community drug distributors or a district Ministry of Health officer to the nature of the survey prior to the study. On the day of the study, mothers gave informed consent by signing (or fingerprinting) a consent sheet.

## 3 Results

### 3.1 Epidemiological surveys: basic demographics

The mean age of children surveyed in Lakes Albert and Victoria was 3·16 years (median of three years, range of nine months to six years) and 3·02 years (median of three years, range of four months to six years), with female to male ratios of 0·64 and 1·11, respectively. The families who participated in our studies were of low socio-economic status (more than 90% of mothers were housewives, farmers or fisherwomen) and 85% used the lake as the main source of water. In both populations, history of previous PZQ treatment was 57·4% in mothers and 6·7% in their children.

### 3.2 Detection of intestinal schistosomiasis

The prevalence of egg-patent intestinal schistosomiasis was 44·3% in children from Lake Albert [GM_W_ = 5·721 (CI_95_ 5·312–6·130) epg] and 16·0% in children from Lake Victoria [GM_W_ = 0·795 (CI_95_ 0·603–0·986) epg], with prevalence of heavy intensity *S. mansoni* infections significantly higher in children from Lake Albert (8·4% v. 1·3%, *P* < 0·0001; Table 1). When considering results pooled from positive criteria on all diagnostic techniques for intestinal schistosomiasis (microscopy, CCA and SEA-ELISA), prevalence of infection was 68·7% for children in Lake Albert, and 58·6% in Lake Victoria. Importantly, the youngest case of egg-patent schistosomiasis was a girl from Lake Victoria (six months of age), while the youngest heavy intensity case (>400 epg) was a boy from Lake Albert (nine months of age). Additionally, positive cases by CCA commenced at six months of age in Lake Victoria and nine months of age in Lake Albert, while the first SEA-ELISA positive children were one year of age in both places.

**Table 1:** Prevalence (and CI_95_) of intestinal schistosomiasis in mothers and children from Lakes Albert and Victoria.

	Lake Albert		Lake Victoria	
Prevalence in % (and CI_95_) of	Mothers (*n* = 125)	Children (*n* = 131)	Mothers (*n* = 120)	Children (*n* = 232)
Egg-positive intestinal schistosomiasis^a^	60·0 (50·8–68·7)	44·3 (35·6–53·2)	29·2 (21·2–38·2)	16·0 (11·5–21·3)
Light intensity (1–100epg)	33·6 (25·4–42·6)	24·4 (17·3–32·7)	21·7 (14·7–30·1)	12·1 (8·2–17·0)
Medium intensity (101–400epg)	14·4 (8·8–21·8)	11·5 (6·6–18·2)	4·2 (1·3–9·5)	2·6 (1·0–5·5)
Heavy intensity (>400epg)	12·0 (6·9–19·0)	8·4 (4·3–14·5)	3·3 (0·9–8·3)	1·3 (0·3–3·7)
Intestinal schistosomiasis^b^	82·2 (74·5–88·4)	68·7 (60·0–76·5)	66·7 (57·5–75·0)	58·6 (52·0–65·0)

a Prevalence of egg-patent infections according to two-day double Kato-Katz smears.

b Prevalence of egg-patent *S. mansoni* infection (Kato-Katz) and/or visual SEA-ELISA positive reaction and/or CCA positives (≥trace).

**Figure 2:**
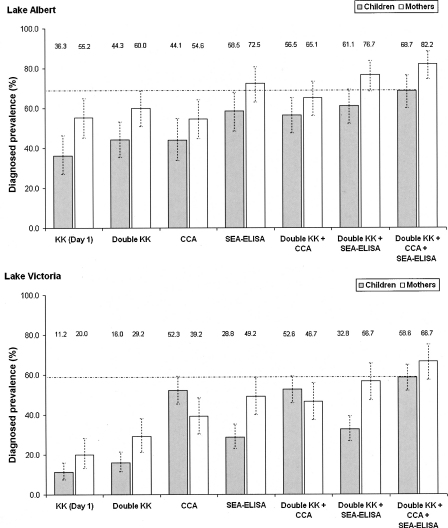
Charts detailing the different prevalence levels of intestinal schistosomiasis, assessed by different diagnostic methodologies, in preschool children (≤6 year olds) and their mothers, from villages on the shores of Lakes Albert (top) and Victoria (bottom), Uganda. CI_95_ around the prevalence are indicated in dashed lines.

The observed intestinal schistosomiasis prevalence according to different techniques, and combinations thereof, are summarised in Figure 2. Diagnosing intestinal schistosomiasis by performing KK smears on two day's stool samples rather than on single stool sample led to a small increase in the prevalence of infection in Lake Albert, and a greater increase in the prevalence of infection in Lake Victoria. CCA as a single diagnostic tool, and compared to KK smears on two day's stool samples, led to small difference in prevalence of *S. mansoni* in families from Lake Albert [mothers (M): from 60·0% to 54·6%; children (C): from 44·3% to 44·1%], while its application in Lake Victoria led to a large increase in the number of individuals considered *S. mansoni*-positive (M: from 29·2% to 39·2%; C: from 16·0% to 52·3%). The use of CCA in conjunction with KK smears from two days stool samples increased the number of *S. mansoni* infections diagnosed, particularly in Lake Victoria and with emphasis on children (M: +60%; C: +229%). Similar trends were observed for SEA-ELISA. Finally, by using all three methodologies (microscopy, CCA and SEA-ELISA), the prevalence of infected mothers rose by 37% in Lake Albert and 128% in Lake Victoria, and the prevalence of infected children rose by 55% in Lake Albert and 266% in Lake Victoria, when compared to those recorded by using egg-positive criteria on KK smears from two day's stool samples as single diagnostic method.

### 3.3 Risk factors associated with egg-patent intestinal schistosomiasis

In shoreline villages of Lake Albert, even though prevalence of egg-patent intestinal schistosomiasis reached 38·1% in children under three years of age, children aged between three and six years were at higher risk of infection than their younger counterparts (OR = 3·4; CI_95_ 1·4–8·2; *P* 0·007). Additionally, children taken to the lakeshore by their mothers when fetching water were five times more likely to be infected than those who are left at home (CI_95_ 1·8–13·6; *P* 0·002). Children whose mothers were infected with *S. mansoni* (egg-patent infection) were 5·5 times more likely to be infected than those whose mothers were free of infection (CI_95_ 2·1–14·4; *P* < 0·001). Children who spent more time in the lake water were more likely to be infected, where every additional minute accounted for a 1% increase in odds of infections (OR = 1·01, CI_95_ 1·003–1·014; *P* 0·003). Children who spent between 30 min and 1 h daily in the lake water were found to be 4·5 times more likely to be infected (CI_95_ 1·5–13·2; *P* 0·008) than those who spent less than 30 min. Likewise, those who spent between 1 h and 4 h, and those who spent more than 4 h daily in the lake water were 6·0 (CI_95_ 2·0–17·7; *P* 0·002) and 11·6 times (CI_95_ 1·1–124·8; *P* 0·046), respectively, more likely to be infected than those who spent less than 30 min. From a geographical perspective, children from the northern villages of Bugoigo and Walukuba were 4·6 times more likely to be infected than those from the southern villages of Tonya and Runga (CI_95_ 2·0–10·2; *P* < 0·001).

In shoreline villages of Lake Victoria, children aged between three and six years were also at higher risk of infection than their younger counterparts (OR = 3·8; CI_95_ 1·8–8·1; *P* < 0·001), with a prevalence of egg-patent intestinal schistosomiasis reaching 8·8% in children under three years of age. Once again, children whose mothers were infected with *S. mansoni* (egg-patent infection) were more likely to be infected than those whose mothers were free of infection (OR = 2·3; CI_95_ 1·1–4·8; *P* 0·035). Children who spent more than 30 min daily in the lake water were 2·5 times more likely to be infected than those who spent less than 30 min (CI_95_ 1·1–5·6; *P* 0·024). Children who knew how to swim were 3·2 more likely to be infected than those who did not know (CI_95_ 1·4–7·1; *P* 0·005). Children who had a toilet available in or around their household were less likely to be infected than those who did not (OR = 0·5; CI_95_ 0·2–1·0; *P* 0·051).

### 3.4 Treatment efficacy and side-effects

PZQ (standard dosage of 40 mg/kg) had a CR of 100·0% (28 children egg-patent at baseline; CI_95_ 87·7–100·0%), with the population prevalence (i.e. inclusive of children who did not receive treatment) of *S. mansoni* egg-patent infections decreasing significantly (*P* < 0·0001) from 16·0% (CI_95_ 11.5–21.3%) to 3·1% (CI_95_ 1·1–6·6%) 21 days after chemotherapy. According to data from CCA diagnosis, PZQ had a CR of 65·1% (83 children positive at baseline; CI_95_53·8–75·2%), with the population prevalence decreasing significantly (*P* < 0·01) from 52·3% (CI_95_ 45·4–59·2%) to 29·4% (CI_95_ 22·9–36·7%) 21 days after chemotherapy. The ERR for PZQ on *S. mansoni* infections was 100·0% (mean infection intensity of those positive at baseline decreased from 139·2 epg to 0·0 epg).

During mass administration of PZQ and ALB in Lake Albert, side-effects directly related to tablet administration (e.g. choking or coughing) were very rare (prevalence <0.1%) and not life-threatening. Twenty-four hours after treatment in Lake Albert 2·6% of children (*n* *=* 1122) reported feeling ill, with abdominal pain (0·9%) and diarrhoea (0·8%) being the commonest symptoms. In Lake Victoria, twenty-four hours after treatment all side-effects investigated, with the exception of headache and lower-back pain, were reported at varying prevalence levels, from 5·1% of children complaining of abdominal pain to 16·1% reporting fatigue. Headache (3·6%), vomiting (9·4%), diarrhoea (10·9%) and urticaria/rash (8·9%) were found at significant levels. By contrast, mothers reported very few children (<1%) with any symptoms present on the day of the 21-day follow-up. For full results see Table 2.

**Table 2:** Percentage (%) of preschool children (≤6 years of age) to report symptoms 24 hours and 21 days after co-administration of albendazole and praziquantel. Note that background incidence of these symptoms (registered prior to treating) has been taken into account when calculating the incidence of actual side-effects. Amelioration is defined as the reduction in prevalence of symptoms after chemotherapy.

Symptoms	24 hours later		21 days later	
	Incidence %	Amelioration %	Incidence %	Amelioration %
Dizziness	10·6	7·4	0·0	7·8
Headache	1·8	47·9	3·6	45·8
Sleepiness	14·7	24·4	1·0	38·5
Fatigue	16·1	19·4	0·5	27·5
Vertigo	13·8	4·6	1·0	6·3
Abdominal pain	5·1	43·3	2·6	55·3
Cramps	11·3	39·2	2·1	43·6
Nausea	8·8	17·1	1·0	20·8
Vomiting	7·8	19·4	9·4	21·4
Lower back pain	0·5	5·1	3·2	6·3
Urticaria/rash	9·2	17·1	8·9	17·7

### 3.5 Treatment administration: description of the extended PZQ dose pole

**Figure 3:**
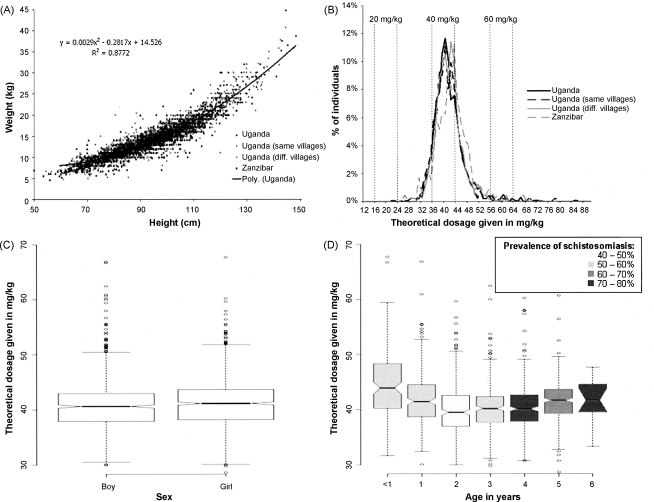
Creating and applying the newly developed extended dose pole for the rapid administration of praziquantel to preschool children (≤six year olds). **A** Distribution of height and weight measurements from Uganda (‘genesis’ population *n* *=* 1046, ‘same villages’ population *n* *=* 1047, ‘different villages’ population *n* *=* 1210) and Zanzibar (*n* *=* 470). A polynomial model was fitted to indicate the correlation between height and weight data from the ‘genesis’ population and then used to predict bodyweight of each subject from height. **B** The distribution of PZQ dosages that would have been given to Ugandan and Zanzibari children if height had been used to predict weight. Vertical dotted lines mark the upper and lower ranges of dosage given in practise if the target dosage is 20, 40 or 60 mg/kg.^28^**C and D** Box-and-whisker plots of theoretical dosages given to the different sexes and ages, respectively, using as test populations those from Uganda (‘different villages’ population) and Zanzibar (total *n* *=* 1680). Shading depicts prevalence of intestinal schistosomiasis at each age according to total diagnostic evidence from the two targeted surveys (see Figure 1), (total population: 18 children were under one, 46 were one, 67 were two, 81 were three, 69 were four, 36 were five and 17 were six years of age). The horizontal bar in the middle shows the median theoretical dosage; both boxes show the interquartile range (25^th^ to 75^th^ percentile); the vertical dashed lines show the minimum and maximum values, or, the presence of outliers (the circles), show 1.5 times the interquartile range (roughly two times the standard deviation); the notches at the ‘waist’ give an impression of CI_95_ around the medians, where boxes whose notches do not overlap have significantly different medians (*P* < 0·05).^49^

**Figure 4:**
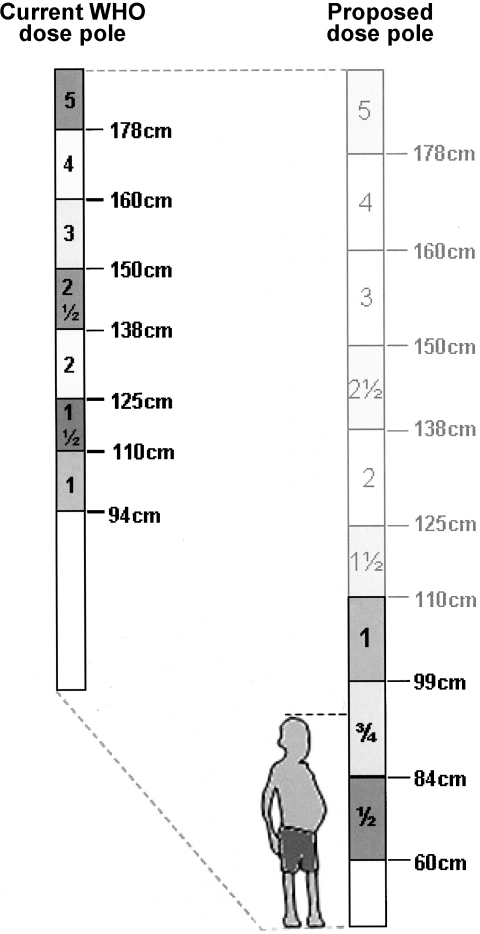
Pictorial representation of the current WHO dose pole for administration of PZQ tablets (at 600 mg each) (left) and the proposed dose pole (right) with two new height thresholds added to allow for treatment of preschool children (≤six year olds): 60–84 cm for one-half and 84–99 cm for three-quarters PZQ table.^19^ The illustrated child needs three-quarters of one tablet. Additionally, the WHO pole's single tablet lower limit has been amended from 94 cm to 99 cm.

Using the equation for the relationship between height and weight based on data from 1046 Ugandan children (*y* = 0·0029x^2^–0·2817*x* + 14.526 in Figure 3A), height thresholds were established corresponding to 7·5 kg, 11·25 kg and 15·0 kg, the weights for one-half (300 mg), three-quarters (450 mg) and one (600 mg) PZQ tablet administration, respectively. The resulting height thresholds values were 60 cm, 84 cm and 99 cm, respectively. For a representation of the current WHO dose pole and the extended version, refer to Figure 4.

Using the equation for the relationship between height and weight to predict the weight of children from the same village, different villages within the same country and children from another African country does not greatly affect the percentage of those who would receive a safe and efficacious PZQ dosage (between 30 and 60 mg/kg). The mean dosage would vary between 40·5 mg/kg for those in the same village to 41·9 mg/kg for those from a different country. The dose pole estimated an optimal dosage in 61·4% (CI_95_ 59·7–63·6%) and an acceptable dosage in 98·6% (CI_95_ 98·1–99·0%) for Ugandan children. For Zanzibari children, the percentages of those that would receive optimal and acceptable dosages were 69·6% (CI_95_ 65·2–73·7%) and 97·6% (CI_95_ 95·9–98·8%), respectively. Only 0·4% of the 3303 Ugandan and 0·9% of the 470 Zanzibari children would have received a sub-curative dosage, and 1·0% Ugandan and 1·5% Zanzibari children would have received a slight over-dosage (Table 3). Figure 3B shows the distribution of dosages on all populations tested and depicts a normal distribution around the target of 40 mg/kg, albeit slightly skewed to the right for Zanzibar indicating a slight tendency to over-dose. Details of the results obtained in the different data sets are presented in Table 3. Additionally, the predicted dosage did not vary significantly between sexes (Figure 3C). As for different ages, the median dosage given to infants (<1 year old) and children aged five years would be slightly higher (*P* < 0·05) than the dosage given to children aged between two and four, albeit still within the safe and efficacious range (Figure 3C and 3D). Very few individuals aged six years were included in the survey (15 of a total of 1680 children), explaining the wide confidence intervals around the median dosage. Prevalence of intestinal schistosomiasis (according to microscopy, rapid diagnostic test and/or serology) increases with age, remaining above 40% at all times.

**Table 3:** Performance of the extended dose pole in estimating PZQ dosages in preschool children (≤6 year olds), from our different datasets (see Methods 2.5, and Figure 3A). A PZQ optimal dose was defined as being between 40–60 mg/kg and an acceptable dose as being between 30–60 mg/kg.^19^.

PZQ dose		Uganda (*n* *=* 1046)	Uganda (*n* *=* 1047)	Uganda (*n* *=* 1210)	Zanzibar (*n* *=* 470)
Dose administered (mg/kg)					
	Minimum	27·2	21·3	24·0	25·3
	Maximum	84·4	89·5	72·3	66·9
	Average (SD^a^)	40·8 (5·4)	40·5 (5·5)	40·9 (4·4)	41·9 (5·7)

No. (%) of individuals receiving dose (%)					
	<30 mg/kg	4 (0·4)	7 (0·7)	1 (0·1)	4 (0·9)
	≥30 & <40 mg/kg	399 (38·1)	409 (39·1)	421 (34·8)	132 (28·1)
	≥40 & <50 mg/kg	577 (55·2)	587 (56·1)	731 (60·4)	301 (64·0)
	≥50 to <60 mg/kg	49 (4·7)	32 (3·1)	52 (4·3)	26 (5·5)
	>60 mg/kg	17 (1·6)	12 (1·1)	5 (0·4)	7 (1·5)

a SD = standard deviation.

## 4 Discussion

In light of these targeted epidemiological surveys, we have further demonstrated the occurrence of intestinal schistosomiasis in preschool children from shoreline villages on two of the major lakes of East Africa. Given our previous observations in Lake Victoria, we have revealed the non-transient nature of this aspect of the disease transmission.^12^ In so doing, we have made explicit the necessity for mass treatment with PZQ for these children.

### 4.1 Intestinal schistosomiasis in young children

Across all surveyed villages, prevalence of egg-patent infection in children was considerable, even more so if we define an infected child as one diagnosed positively by any of the diagnostic tools used–microscopy, CCA and SEA-ELISA–with overall prevalence of 62.3% (CI_95_ 57.1–67.3), consistent with previous results for preschool children and similar to that reported for school-aged children from the same districts.^12^, ^17^, ^23^ Although egg-patent intestinal schistosomiasis was present in very young children (youngest aged six months), with heavy intensity infections observed in children as young as nine months of age, children aged between three and six years appeared to have raised intensities of infection. This observation is likely representative of the cumulative and increasing water contact behaviour of the child, combined with the maturation and increasing egg-laying capacity of schistosome worm pairs within the child linked with advancing years. It should be noted, however, that mothers have an important role in influencing their child's infection status, particularly during the first 24 months of life (i.e. likely when initial exposure(s) to water containing schistosome cercariae commences), when the child is regularly taken to the lakeshore during household chores and child-bathing.

These observations support a predominantly bimodal profile of exposure, from a spectrum of passive and active processes, in this younger age-class, as suggested by Stothard and Gabrielli.^14^ For example, children aged three and below are much more dependent on their mother's movements and child-bathing practices, so any contact with freshly drawn lake water is largely an involuntary, or passive action of the child. By contrast, in slightly older children, who have greater mobility and independence, voluntary actions increase their water contact at lakeside points, whilst they are still vulnerable to any other ongoing involuntary exposures.^14^

A heterogeneous distribution in parasite prevalence was observed across lakeshore villages. In Lake Albert, for example, the two northern villages–Bugoigo and Walukuba–had significantly more intestinal schistosomiasis than the southern villages, even though they were only separated by 15–30 km of lakeshore (refer to Figure 1). A plausible explanation for this focality, typical of schistosomiasis, is differing abundance of snail hosts in the southern villages which were located on shorelines with predominately more wave exposure. Freshwater snails of the genus *Biomphalaria* are very sensitive to local aquatic environments and only certain locations on the lakeshore provide the optimal conditions.^36^, ^37^ Furthermore, on both lakeshores there was a household, or familial, aggregation of intestinal schistosomiasis indicating disease focality or ‘patchiness’ even within a single village. Those households with heightened levels of exposure, and thus more vulnerable to infection, are likely located close to niches highly infested with the snail hosts.

With preschool children and infants typically accounting for the widest age-bracket on the population pyramid of low and medium income countries, the numbers of those at risk of neglected tropical diseases in the ‘bottom billion’ is significantly increased.^38^ Importantly, the significance of schistosomiasis in such young children may be presently underestimated, as synergisms with concurrent infections (e.g. malaria), or predisposition towards exposure and infection of others (e.g. HIV), are still to be determined and may start much earlier in life than previously thought. 39, 40

### 4.2 Diagnosing intestinal schistosomiasis in preschool children

Given the heterogeneity of the epidemiological landscape of Uganda, a future challenge for mass treatment campaigns is to ensure that PZQ is administered in the most cost-effective and evidence-based way. Thus before mass drug administration takes place, some attention should be given to the rapid identification of those communities where intestinal schistosomiasis in preschool children is common. From our current perspective, it can be safely assumed that mass treatment is urgently needed in villages on the immediate shorelines of these lakes without additional surveying, but moving inland and farther away from the lake, or outside these areas, there is a need for rapid assessment methods. From our experiences, the most favourable diagnostic technique for *S. mansoni* appears the CCA urine-dipstick, given the evidence of the test's satisfactory performance in school-aged children, its cost-effectiveness (cost: £1·60; diagnosis time: 20 min), ability to detect non-egg-patent infections and ease of use by staff with minimal training.^26^ On the other hand, even at £1·60, CCA remains comparatively more expensive than KK smear examination, which performed reasonably well in high prevalence/infection intensity scenarios, such as the Lake Albert shoreline. Given that stool microscopy is routinely conducted in Uganda, we suggest that KK smears from a single stool sample, followed by statistical scaling, is an appropriate technology to make a pragmatic estimate of the local prevalence of intestinal schistosomiasis in preschool children.

Although CCA and SEA-ELISA, as single diagnostic tools, tended to identify increased numbers of children positive for *S. mansoni* compared to double-sample KK smears at both lake locations, this difference was more evident in Lake Victoria: +227% (from 16·0% to 52·3%) for CCA and +80% (from 16·0% to 28·8%) for SEA-ELISA. This together with the fact that age is a factor associated with infection intensity, could be an indication that in Lake Victoria, for example, heavier infections are much slower to accrue than in Lake Albert reflecting transmission heterogeneities between lacustrine environments which is also supported by differences in infection intensities of the mothers. Recent research has also identified that parasite populations between lakes are genetically distinctive, which may have some bearing upon egg production patterns and immuno-pathogenic profiles.^41^

### 4.3 PZQ and the young child

Although a relatively small sample size was used to calculate CRs and ERRs, the results fall within the ranges found in the existing literature with good parasitological performance.^42^, ^43^, ^44^, ^45^ Administration of PZQ in the young child resulted in some putative side-effects, although many had resolved by 24 hours after treatment and even fewer were present 21 days after treatment, evidence of their mild and transient nature, and in accordance with previous observations. An obvious caveat for our data is the fact that symptoms were being reported by the mother on behalf of the child.^46^ Interestingly, mothers reported significantly more side-effects one day after they received treatment, particularly dizziness (percentage difference = +10%), sleepiness (+15%), fatigue (+16%), vertigo (+14%) and cramps (+11%), than their children, possibly due to the higher prevalence and mean intensity of *S. mansoni* infections recorded in the mothers.

These results and observations on the risk of infection in the very young child, point to the necessity of producing a PZQ syrup formulation at low cost but it should be remembered that these formulations tend to have shorter shelf-lives and thus pose a greater challenge for field logistics than tablets. In addition, using syrup requires an entirely different dosing pole. Although time-consuming, crushing the tablets with flavoured juice is practicable, with minimal impact on the efficacy of the drug, and an extended dosing pole is now available. As an additional future refinement, PZQ tablets should be manufactured with markings to allow easy division into four quarters for more precise dose estimation when treating children weighing between 7·5 and 11·25 kg. Finally, administration of PZQ to young children will be made easier after the development of more palatable PZQ tablets with the exclusion of the inactive isomer that gives the drug a bitter taste, though we have seen that many younger children do not immediately recognise the unusual taste of PZQ.^47^

Two new height intervals have been added to extend the PZQ dose pole: 60–84 cm for one-half tablet and 84–99 cm for three-quarters of one tablet. Additionally, as ascertained using a dataset composed largely of preschool children, the current WHO pole's single table lower limit was adjusted from 94 cm to 99 cm.^19^ Our extended PZQ dose pole was very accurate within Uganda as well as in Zanzibar (prevalence levels of acceptable dosages estimated were 98·6% and 97·6%, respectively). Although we have demonstrated our pole would be exportable to other communities outside of Uganda (e.g. Zanzibar, Tanzania), we suggest the evaluation of these new divisions by other African Ministries of Health. Additionally, further safety and efficacy tests need to be conducted before implementing a formal update to the current WHO dose pole, consequently including children less than four years of age. In the absence of a Paediatric Investigation Plan (PIP) or equivalent (Paediatric Research Equity Plan; PREA) for PZQ, the international community needs to consider ways in which drug safety could be sensibly assessed perhaps in a manner similar as to how the inclusion of pregnant women in mass treatment campaigns was advocated.^18^ It is worth noting that interventions targeting STH infections in preschool children are out-performing, in terms of treatment coverage, school-based interventions, so if the above steps are set in place, control of schistosomiasis in these young children could achieve such programmatic performance within the foreseeable future.^48^

## 5 Conclusion

We call for the immediate inclusion of preschool children in the Ugandan national schistosomiasis control programme and, to facilitate the administration of PZQ, our extended dose pole can be applied. In so doing, we take a crucial step towards addressing this issue in Uganda and now draw attention to the present health inequity elsewhere in sub-Saharan Africa.

## Authors’ contributions

JRS, NBK and JCSF contributed to the design of the studies; JCSF, MB, JP, MD, FK, DR, and JRS participated in data collection; JCSF, JP, MD and MB participated in data entry, JCSF, MB, NBK, AM and JRS participated in data interpretation. JRS and NBK coordinated the studies and MD and FK supervised the follow-up of patients. JCSF conducted analysis of data. JCSF, JP, MD, MB, DR, NBK, FK, AM and JRS participated in the preparation of the report and approved the final version. JCSF and JRS are joint guarantors of the paper.

## Funding

This work was supported by a project grant awarded to JRS by the Wellcome Trust and umbrella funding from the EU under the auspices of CONTRAST (project number 032203).

## Conflicts of interest

The authors report no conflict of interests. The authors alone are responsible for the views expressed in this article, which might not necessarily reflect the opinion or policy of their employing institutions.

## Ethical approval

The Ugandan National Council of Science and Technology and the London School of Hygiene and Tropical Medicine, UK, granted ethical approval for these studies (application nos. LSHTM 06·45 and LSHTM 5538·09).
